# The Old and New Visions of Biased Agonism Through the Prism of Adenosine Receptor Signaling and Receptor/Receptor and Receptor/Protein Interactions

**DOI:** 10.3389/fphar.2020.628601

**Published:** 2021-01-29

**Authors:** Rafael Franco, Rafael Rivas‐Santisteban, Irene Reyes-Resina, Gemma Navarro

**Affiliations:** ^1^ Department Biochemistry and Molecular Biomedicine, School of Biology, University of Barcelona, Barcelona, Spain; ^2^ Centro de Investigación en Red, Enfermedades Neurodegenerativas (CiberNed), Instituto de Salud Carlos iii, Madrid, Spain; ^3^ RG Neuroplasticity, Leibniz Institute for Neurobiology, Magdeburg, Germany; ^4^ Department of Biochemistry and Physiology, School of Pharmacy, University of Barcelona, Barcelona, Spain

**Keywords:** cAMP, MAPK pathway, adenylyl cyclase, GPCR, tetramer, heteromer, receptor-receptor interactions, functional selectivity

## Abstract

Biased signaling is a concept that has arisen in the G protein-coupled receptor (GCPR) research field, and holds promise for the development of new drug development strategies. It consists of different signaling outputs depending on the agonist’s chemical structure. Here we review the most accepted mechanisms for explaining biased agonism, namely the induced fit hypothesis and the key/lock hypothesis, but we also consider how bias can be produced by a given agonist. In fact, different signaling outputs may originate at a given receptor when activated by, for instance, the endogenous agonist. We take advantage of results obtained with adenosine receptors to explain how such mechanism of functional selectivity depends on the context, being receptor-receptor interactions (heteromerization) one of the most relevant and most studied mechanisms for mammalian homeostasis. Considering all the possible mechanisms underlying functional selectivity is essential to optimize the selection of biased agonists in the design of drugs targeting GPCRs.

## Introduction

Biased signaling consists of different signaling outputs depending on the agonist chemical structure. The concept has taken hold in the field of G protein-coupled receptor (GPCR) research and has opened up new perspectives for therapeutic drug development. The underlying idea is that a given agonist biased towards a particular signaling may be therapeutic while another agonist biased towards activating an alternative pathway may not be helpful, and may even be harmful.

Biased agonism is an attractive concept to try to get agonist use off the ground in clinical practice. At present, agonists have by far less potential than antagonists. Usually, endogenous agonists approved as therapeutic drugs are used in acute conditions and during short times. In contrast, antagonists may be used in a chronic regime. The classical example is epinephrine that is used as adrenergic agonist to save lives in critical situations (e.g., anaphylaxis) whereas beta-adrenergic blockers/antagonists are used for a variety of diseases in both acute and chronic regimes. In the purine field, adenosine is used in bolus administration to combat paroxysmal tachycardia whereas the adenosine A_2A_R antagonist, istradefylline (Nouriast™ in Japan; Nourianz™ in the United States), has been approved for chronic use in the therapy of Parkinson’s disease (Pinna et al., 2007; Simola et al., 2008; Jenner et al., 2009; Mizuno and Kondo, 2013; Kondo et al., 2015; Navarro et al., 2015).

Two complementary points of view are needed to underline the mechanisms underlying differential signaling arising from a given GPCR. In a previous paper we already made a distinction between biased signaling and biased functionality (Franco et al., 2018). Here we will provide more information on the possibility that biased signaling arises from different compounds acting on the same receptor but, also, on the possibility that biased signaling arises from the same agonist acting in the same receptor but expressed in a different context. By different context we mean that a given GPCR may be expressed in different cells coupled to different proteins, not only to different G proteins but to other receptors, to scaffolding proteins, etc.

## The Most Accepted Mechanism to Explain Biased Signaling

The resolution of the structure of various GPCRs and the molecular dynamics of macromolecules in aqueous solutions give indications as to how GPCR-mediated signaling occurs. Binding of the agonist to the orthosteric site leads to significant structural rearrangements that are transmitted to the coupled G protein and allow signaling (Westfield et al., 2011; Masureel et al., 2018).

**FIGURE 1:**
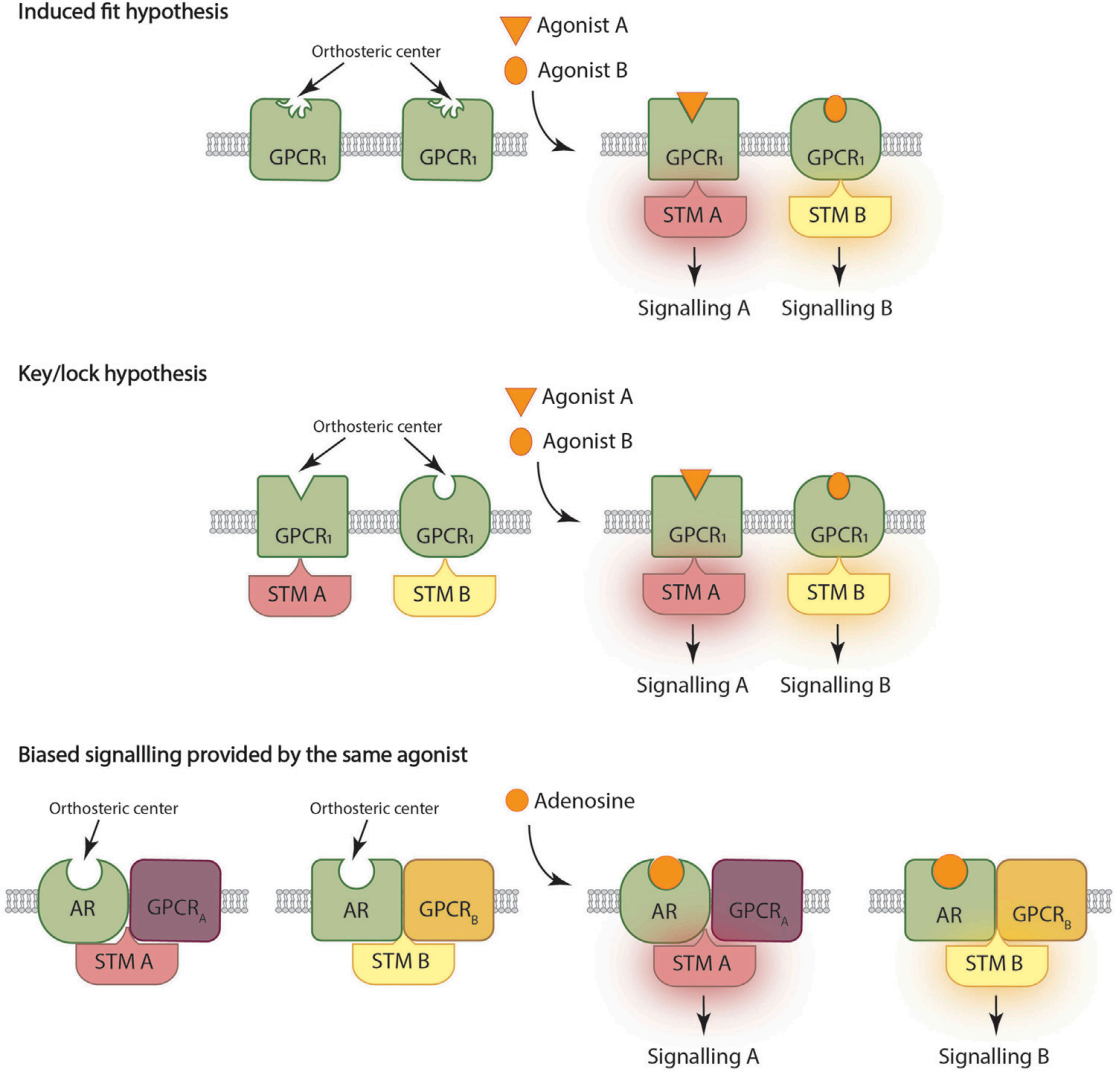
Three ways to deliver biased signaling. Top: G protein coupled receptor (GPCR) orthosteric center of the GPCR has a loose conformation that is fixed upon agonist binding, thus allowing coupling and engagement of the signal transduction machinery (STM). Center: There are different conformational states of a given GPCR with structurally different orthosteric sites; each agonist preferentially binds to a given conformation thus preferentially engaging a given STM. Bottom: A given ligand acting on a given GPCR may lead to different signaling outputs depending on the context of the receptor. The case of heteromer formation is exemplified with a given adenosine receptor (AR) able to interact with GPCR_A_ or with GPCR_B_.

It is not necessary to be very specific with the details to explain the basis of the most accepted mechanism underlying biased signaling. In fact, assuming that GPCRs have a loose orthosteric center, the binding of structurally different chemicals to the site can result in different conformations (Figure 1 up). Said different conformations will couple differently to the signaling machinery, thus providing different signaling outputs. The agonist/receptor interaction would be similar to the so-called induced fit in the case of a substrate that interacts with the active site of an enzyme (Urban et al., 2007; Kenakin and Miller, 2010) (Kenakin, 2009; Kenakin, 2011; Kenakin and Christopoulos, 2013).

Following the analogy with substrate/enzyme interaction, there is another point of view which is that the cell surface receptor is in different conformational states while waiting for the arrival of the agonist. Similar to the key/lock idea (Figure 1 center), each of these conformational states would have a different lock and each agonist would interact more strongly (i.e., with more affinity) with some conformations than with others (Costa-Neto et al., 2016; Michel and Charlton, 2018).

In summary, in the classical view each agonist favors a specific signal transduction and that this may be due to two conceptually different mechanisms. One is by assuming different receptor states due to pre-coupling to signaling mechanisms and each chemical structure preferentially binding to a given state, thereby preferentially engaging such particular signaling pathway. The second is by assuming the GPCR in a given state that, after agonist-induced conformational changes, would lead to a receptor prone to interact to (and engage) a particular signaling machinery.

## The Alternative Mechanism to Approach Biased Signaling. How the Endogenous Agonist May Provide Functional Diversity

Biased agonism fits into a more general framework, called functional selectivity. A given GPCR may provide different signaling outputs depending on the context. In other words, functional selectivity may be afforded using a single agonist. Yet another way to express the idea is that the endogenous agonist (hormone/neurotransmitter) will give rise to different signals depending on the cell/tissue and the general pathophysiological state.

We argue, as suggested elsewhere (Franco et al., 2018), that biased signaling does not require a biased agonist, that is, that the endogenous agonist may engage different signaling pathways depending on the cell context. In short, it would be the functional unit itself, made up of the receptor and the direct receptor/receptor and receptor/protein interactions, which is coupled to a certain signaling machinery. Consequently, cells will respond according to the coupling assigned to the specific structure of the functional unit and the existence, or not, of more than one functional unit.

Below we will present some examples of differential functional selectivity provided by an endogenous agonist (Figure 1 bottom). Let us first describe the classic case discovered by Susan George and her colleagues working with dopamine receptors. According to IUPHAR, the cognate G proteins for the D_1_ and D_2_ receptors are, respectively, G_s_ and G_i_ (Alexander et al., 2019). However, D_1_ and D_2_ can interact to form D_1_-D_2_ receptor heteromers that do not couple to G_s_/G_i_ but to G_q_. Coupling to G_q_ allows dopamine to activate not only cAMP- but also calcium-related mechanisms (Lee et al., 2004; Rashid et al., 2007; Perreault et al., 2015; Perreault et al., 2016). The controversy that arose about the appearance of such complexes in primates has been resolved by finding that about 18% of the neurons of the striatum of *Macaca fascicularis* express the D_1_-D_2_ receptor heteromers (Rico et al., 2016). In fact, there are neurons in different parts of the central nervous system that expressing those heteromers provide a long-suspected link between dopaminergic neurotransmission and calcium signals.

Dopamine D_1_ receptors can also form heteromers with histamine receptors, whose exact role in the central nervous system has yet to be fully clarified. Interestingly, the formation of D_1_ and the histamine H_3_ receptor heteromer is required for histamine to activate the mitogen-activated protein kinase (MAPK) signaling pathway. Surprisingly, it appears that D_1_ receptors within this heteromeric context bind to G_i_ rather than its cognate G protein, G_s_. (Ferrada et al., 2009).

A final example we provide here is related to GPCRs that regulate intraocular pressure. Melatonin receptors form functional complexes with α_1_-adrenergic receptors, which involve the C-terminal tail of the latter. Surprisingly, activation of α_1_-adrenergic receptors in this particular heteromeric context does not lead to changes in cytoplasmic levels of Ca^2+^ but of cAMP. Once again, the heteromeric context leads to a change, from G_q_ to G_s_, in the G protein coupling (see Figure 1 bottom). Glaucoma coursing with elevated intraocular pressure is correlated with a decreased expression of the complexes in stromal cells (Alkozi et al., 2019; Alkozi et al., 2020). Whether this fact is a cause or a consequence of the disease, the melatonin-adrenergic heteromers arise as targets for fighting the disease.

## Biased Signaling Under the Prism of Results Derived From Adenosine Receptor Signaling Characterization

Four are the adenosine receptors identified so far in mammals: A_1_, A_2A_, A_2B_, and A_3_. The cognate G proteins for the A_1_ and the A_3_ are of the G_i_ type and the cognate G proteins for the A_2A_ and the A_2B_ are of the G_s_ type. Via G protein-mediated signaling or via the ßγ subunits of G proteins, activation of adenosine receptors may activate the mitogen-activated protein kinase (MAPK) pathway. Also ß-arrestin recruitment may lead to receptor internalization and intracellular signaling (see Borea et al., 2018 for review).

Recently, we have performed a classic study of biased agonism using one of the four adenosine receptors, the A_2A_, expressed in a heterologous system. In addition to identifying two chemical structures, PSB-0777 and LUF-5834, that behaved differently from the rest of the agonists, we noticed that removing part of the receptor’s C-terminal tail does not qualitatively change the results (Navarro et al., 2020). This finding was unexpected as the long C-terminal end of the receptor is potentially interacting with some components of the signaling machinery. Interestingly, removal of the C-terminal tail of the A_3_ receptor is dispensable for its capability to recruit ß-arrestins (Storme et al., 2018).

It is now suspected that the four adenosine receptors, A_1_, A_2A_, A_2B_, and A_3_, can interact with each other. The A_1_-A_2A_, A_1_-A_3_, and A_2A_-A_2B_ interactions have already been described (Ciruela et al., 2006a; Hill et al., 2014; Hinz et al., 2018; Lillo et al., 2020). The interaction between the adenosine A_2A_ and A_1_ receptors was identified several years ago (Ciruela et al., 2006a) and the functional role of the complex has been well understood ever since (Ciruela et al., 2006b; Cristóvão-Ferreira et al., 2013; Lin et al., 2020). A recent review on structure and function of adenosine receptor heteromers is available (Franco et al., 2021).

Adenosine leads to marked biased signaling based on the heteromeric context, even considering only interactions between adenosine receptors. On the one hand, signaling mediated by the A_3_ receptor is blocked if A_2A_R is co-expressed and A_2A_-A_3_ receptor heteromers are formed. A_2A_ receptor antagonists abrogate the blockade, thus providing a novel approach to the development of drugs that target the heteromers of the A_2A_-A_3_ receptor. On the other hand, the expression of the A_2B_ receptor blocks signaling through the A_2A_ receptor. This finding raises several questions, as the A_2A_ receptor has a much higher affinity for adenosine than the A_2B_. The actual physiological significance of this interaction is under close scrutiny, although the A_2A_-A_2B_ receptor functional complex has already been shown to be relevant in aging and obesity (Gnad et al., 2020).

Remarkably, the A_1_-A_2A_ receptor heteromer adds an additional dimension to functional selectivity. In fact, the signal is biased not only by the endogenous agonist, but also by its concentration. As we often mention, this complex is an adenosine concentration sensor. At concentrations at which only the A_1_ receptor is occupied by adenosine, only G_i_-mediated signaling is observed, with G_i_ being the cognate protein of the A_1_ receptor. In contrast, when the adenosine concentration increases and the A_2A_ receptor is occupied, only G_s_-mediated signaling originates in the heteromer, with G_s_ being the cognate protein of the A_2A_ receptor. The mechanistic molecular basis of such a phenomenon has been fully elucidated and, more importantly, it is the C-terminal tail of A_2A_ that is relevant for blocking the partner (A_1_) receptor function (Navarro et al., 2016; Navarro et al., 2018).

In summary, biased signaling is produced by the endogenous agonist, adenosine, depending on the context of the target receptor, even depending on the adenosine concentration itself. It should be noted that the panorama of functional diversity that adenosine can cause is not limited to the interaction between adenosine receptors, but extends to the complexes that adenosine receptors establishes with other GPCRs or other proteins (see (Ginés et al., 2001; Agnati et al., 2003; Burgueño et al., 2003; Franco et al., 2005; Ciruela et al., 2006b; Fuxe et al., 2007; Navarro et al., 2014) for review).

## Conclusion

The hopes placed on the biased agonism to give an extra boost to the drug discovery are not being fulfilled. For the above reasons, a biased agonist may provide a benefit in a given setting but provide a detrimental effect in other receptor settings and thus not be useful in therapy. Also relevant is how to reliably measure the output of the signaling pathway one wants to target; in fact, different assays claiming to evaluate the same pathway may produce a different result, some suggesting bias, some not. One wonders if it would be more proactive to skip *in vitro* pharmacological assays and test different agonists for their efficacy and safety in in vivo disease models. To date, trying to decipher the mechanism underlying functional selectivity for a given GPCR is challenging. Without this information, it is virtually impossible to optimize the selection of biased agonists for drug development. Therefore, it seems necessary to carry out an investigation aimed at knowing both 1) what is the signaling pathway to target 2) how to reliably measure the pathway output and 3) what is the status of the target GPCR. Status means identifying the proteins/receptors that interact with the target GPCR and how that macromolecular complex is specifically coupled to the signaling machineries.

## Data Availability Statement

The raw data supporting the conclusions of this article will be made available by the authors, without undue reservation.

## Author Contributions

RF planned the contents and discussed them with RR‐S, IR-R, and GN. RF, and GN wrote a first draft. RR‐S, and IR-R made significant additions to the draft. IR-R made the figure. All authors have approved the submitted version.

## Funding

This research was funded by the Spanish Ministry of Economy and Competitiveness (grants: SAF2017-84117-R and RTI2018-094204-B-I00; they may include European Regional Development -FEDER- funds) and the Alzheimer’s Association (grant: AARFD-17-503612). The laboratory of the University of Barcelona is considered of excellence (grup consolidat #2017 SGR 1497) by the Regional Catalonian Government, which does not provide any specific funding for reagents or for payment of services or for Open Access fees.

## Conflict of Interest

The authors declare that the research was conducted in the absence of any commercial or financial relationships that could be construed as a potential conflict of interest.
